# Shared decision making on mode of delivery following a prior cesarean delivery in Dar es Salaam, Tanzania

**DOI:** 10.1371/journal.pone.0291809

**Published:** 2023-10-26

**Authors:** Zainab Hassan Yussuph, Fadhlun M. Alwy Al-beity

**Affiliations:** Department of Obstetrics and Gynecology, School of Medicine, Muhimbili University of Health and Allied Sciences (MUHAS), Dar es Salaam, Tanzania; Werabe University, ETHIOPIA

## Abstract

**Background:**

Shared decision-making between clinicians and pregnant women with prior cesarean on the subsequent mode of delivery improves trial of labor rates, and reduces the number of repeat cesarean sections and their related complications. However, this practice is insufficient worldwide and the factors influencing it are still unknown. The study aimed at determining the proportion of pregnant women involved in shared decision-making and its associated factors in Dar es Salaam.

**Methods:**

A cross-sectional analytical study among 350 pregnant women with one prior cesarean section. Data was collected using a structured questionnaire and SPSS 23 was used for analysis. A score of 80 or higher on the nine-item Shared Decision-Making Questionnaire (SDM-Q9) was used to calculate the proportion of women, and the associated factors were obtained using a logistic regression model. P value of < 0.05 was considered significant.

**Results:**

The proportion of pregnant women involved in shared decision making was 38%. Factors that were significantly associated with sharing decision making were; having low level of education (AOR 0.55 95% CI 0.33–0.91), being married/having partner (AOR 2.58 95% CI 1.43–4.63), having a companion who had active participation (AOR 3.31 95% CI 1.03–10.6) and being familiar with the clinician (AOR 5.01 95% CI 1.30–19.2).

**Conclusion:**

To promote practice of shared decision making in our setting, encouragement of socially vulnerable pregnant women’s participation in decision-making by health care professionals, encouragement of companion participation during antenatal care and promotion of personal continuity of care to improve familiarity to clinicians are needed.

## Introduction

Worldwide, the rate of births by Caesarean section (CS) is rising, varying from 5% in Sub-Saharan Africa to 42.8% in Latin America and the Caribbean [1]. The rate of CS in public healthcare settings was 15.6% in Ghana and 64.2% in private settings, in Sub-Saharan Africa [2]. In Tanzania, the population-based CS rate rose from 2% to 6% over the past two decades (1996–2016) [3]. Alarmingly, facility-based studies document a 30-percent point rise in the CS rate in tertiary level centers over three years, from 19 to 49% between 2009 and 2011, with the most prevalent cause being the prior cesarean section [4, 5]. Additionally, recurrent CS-related morbidity and mortality have been also been increasing [4].

Uterine rupture, placenta previa, placenta accrete spectrum, hysterectomy, intra-abdominal adhesions, and bladder injury are among the morbidities linked to recurrent CS [1]. Current practices encourage women with one prior cesarean delivery to attempt vaginal birth with their subsequent pregnancies [6–8]. It is anticipated that the trial of labor after cesarean (TOLAC) will lower the high rates of cesarean deliveries that have been documented globally and their associated complications [1]. However, the national hospital in Tanzania recorded a very low rate of TOLAC in 2007–0.14% [9].

The main causes of the decline in TOLAC are clinicians’ fears of TOLAC-related adverse effects and of being held accountable or facing legal action from the medical community [10–13]. Currently, it is advised that the choice of delivery method for eligible pregnant women with a history of CS be made through shared decision-making (SDM), during which the pregnant woman should be informed of the risks and benefits of the two delivery options (TOLAC and repeat CS) [6–8].

SDM is recommended so as to support patient autonomy, promote the good clinical practice, and improve patient experiences [14, 15]. Studies conducted in various contexts found that SDM increased TOLAC rates [16, 17], decreased repeat CS by 40%, and improved decision-making and quality of care [16, 18]. Unfortunately, SDM is not consistently practiced globally [16–22], and barriers to its use include low women’s social economic status and education level as well as clinicians’ fear of unfavorable outcomes [10–13, 22–27]

There is a dearth of knowledge on SDM practices on the delivery method for pregnant women who had one prior CS and the obstacles that lead to fewer SDM practices in the Tanzanian context. The study’s findings will provide an understanding of current SDM practices and suggest potential directions for stepping up SDM implementation efforts in our environment. These would potentially increase TOLAC, lower our setting’s high rates of repeat CS, and enhance patient care.

## Materials and methods

### Study design, setting and population

A cross-sectional analytical study was conducted from August to December 2020 in the antenatal clinics of Amana, Temeke, and Mwananyamala hospitals. The three hospitals are regional referral hospitals located in Dar es Salaam, which has a population of over 6 million and is the major commercial hub in Tanzania. The hospitals recorded CS rates of 35%, 43%, and 36% in the second half of 2020, respectively. Women with prior CS deliveries in the past are treated at referral hospitals because they are considered to be at high risk.

We included all women with one prior CS scar, a singleton pregnancy in cephalic presentation at ≥34 week’s gestation and who had two or more antenatal care contacts at the study facility. This was considered adequate contact time to have a dialogue on SDM. Women who had contraindications to subsequent vaginal deliveries using local guideline such as prior classical scar, mal presentation and twins were excluded from the study.

### Sample size

Using a single population proportion formula at 95% power and an estimated proportion of 30.6% [19] from a nearby country, Kenya, we calculated the sample size to be 350 women with prior CS deliveries. The selection was then taken using probability proportion to size sampling from the three facilities. At each facility participants were recognized with the aid of a checklist, and consecutive sampling was used to recruit the participants as the eligible participants were few.

### Study tool

Structured questionnaire in the local language: Swahili was used. The questionnaire was adapted from original German SDM-Q9 [28] and had two parts: part one for participant’s demographic and socio-economic; age, education level, employment status, marital status and payment category. Part 2 had 9-item SDM scale (SDM-Q9). The SDM-Q9 questions used Likert scale with 5 points: ranging from ‘completely disagree’ to ‘completely agree’ scoring 0 to 5. Participants were instructed to select one option that best matched their perceived involvement in each step of the SDM process during the last six months of ANC contacts. Familiarity was defined by pregnant woman personally knowing her clinician or by being attended by same clinician on a prior visit and graded by Likert scale and companion participation was when the person (husband/relative/friend) accompanying the pregnant woman to the clinic actively took part or asked about the modes of delivery, benefits and harm. The questionnaire was paper-based and interviewer administered, and was pretested among 20 eligible women at a similar high-volume health facility.

### Data collection procedure

A pair of research assistants was stationed at antenatal clinics of each of the study facility. Eligible women were identified and recruited at registration. Those who consented were interviewed in a quiet room after completion of clinical care services.

### Data analysis

Data was cleaned and checked for completeness and consistency. Variables were coded into SPSS Version 23 where analysis was done. The descriptive variables were categorized as: Education; no formal/primary education and secondary school and above, age; 20–34 years and >35 years. Marital status; married/cohabiting and single/divorced/widowed, Employment status; employed/self-employed and no employment, payment category; health Insurance/private and cost sharing. Participant and companion participation; participated and not participated and familiarity with clinician; familiar (knowing the clinician very well, well, rather well) and not familiar (not well, not well at all).

The variables were summarized using frequencies and percentages. For SDM: The raw score obtained from 9-item SDM scale which had a maximum score of 45 was transformed as suggested by original SDM-Q9 [28] by multiplying the score to 20/9 to get a score of SDM ranging from 0–100 and score of ≥80 was used to obtain proportion of women with SDM. Measures of association were calculated using cross tabulation and binary logistic regression. The association between the categorized age, education level, marital status, occupation, mode of payment, active participation and familiarity to clinicians as independent variables and SDM as dependent variable was obtained by multivariable logistic regression model at P-value of <0.05.

### Ethical issues

The study was reviewed and received ethical permit from MUHAS Ref No MUHAS-REC-06-2020-283. All participants consented. Those who were eligible received information about TOLAC.

## Results

During the study period, 1940 pregnant women attended antenatal clinic at the facilities, approximately 24.9% (484/1940) had one prior CS scar. Three hundred and sixty were eligible for recruitment, ten-women did not consent on fear of long waiting time, and 350 women were interviewed.

### Demographic and economic characteristics of the study population

Majority of women (93%) with one CS scar were between 20–34 years of age. Mean age was 28.16 ±4.27. Two thirds of the participants were living in union, were employed and had secondary education or above as shown in Table 1. Approximately 58% had private insurance.

**Table 1:** Demographic and economic characteristics of the study population (N = 350).

Variable	Frequency n (%)
**Age group**	
20–34	328 (93.71)
≥ 35	22 (6.29)
Mean age	28.16 ±4.27
**Marital Status**	
Single/Widowed/divorced	80 (22.86)
Married/Cohabiting	270 (77.14)
**Education level**	
No formal & Primary education	107 (30.57)
Secondary & above	243 (69.43)
**Occupation**	
Unemployed	103 (29.43)
Employed/self employed	247 (70.57)
**Payment category**	
Private / Insurance	204 (58.29)
Cost sharing	146 (41.71)

### Proportion of SDM and factors associated with SDM among pregnant women

The proportion of pregnant women with one prior caesarean who were involved in SDM on their mode of delivery for the index pregnancy at all facilities was found to be 38% (133/350).

As shown in Table 2, women with no education and primary education had less odds to be involved in SDM compared with those with secondary education and above (AOR 0.55, 95% CI (0.33–0.91), with P-value = 0.02). Women who were married/cohabiting and women with companion who had active participation were almost 3 times more likely to be involved (AOR 2.58, (95% CI: 1.43–4.63), p-value 0.001) than women who were single/widowed/ divorced and those who had no companion participation. Participants with familiarity to clinicians had 5 times the odds to be involved in SDM 2.58(95% CI: 1.43–4.63) P-value of 0.02.

**Table 2:** Binary logistic regression analysis showing factors associated with SDM.

Variable	Univariable analysis		Multivariable analysis	
	COR (95% C.I)	P-Value	AOR (95% C.I)	P-Value
**Education Level**				
Secondary & Higher education	1			
No formal & primary Education	0.56(0.34–0.92)	**0.022**	0.55(0.33–0.91)	**0.022**
**Occupation**				
Unemployed	1			
Employed	1.98(1.12–3.05)	**0.015**	1.48(0.83–2.63)	0.176
**Marital status**				
Single/widowed/divorced	1			
Married/cohabiting	2.56(1.43–4.55)	**0.001**	2.58(1.43–4.63)	**0.001**
**Payment category**				
Cost sharing	1			
Private/Insurance	1.61(1.03–2.52)	**0.035**	1.33(0.83–2.15)	0.23
**Active participation**				
No	1			
Yes	12.27(4.80–31.35)	**<0.0001**	1.89(0.44–8.01)	0.39
**Companion participation**				
No	1			
Yes	7.07 (2.58–19.39)	**<0.0001**	3.31(1.03–10.64)	**0.04**
**Familiarity with clinician**				
No	1			
Yes	6.42(3.08–13.39)	**<0.0001**	5.01(1.30–19.25)	**0.02**

## Discussion

Nearly a quarter of women who attended antenatal clinics at the referral facilities had one prior CS and lower than half were offered SDM for their index delivery method. Women who reported to have received SDM were more educated, married, or living with a partner, had a companion with active participation in antenatal care, and were familiar with the clinician providing the care.

The study found lower than half of pregnant women with one prior CS was involved in SDM, similar to studies in other various regions [19, 20, 27, 29]. On the other hand, SDM was high in a hospital associated with a tertiary university in Peru [30]. The study in Peru was done among women with high health literacy, which positively affects SDM. This could account for the difference.

Women with less education had fewer odds of participating in SDM than women with more education. The findings are consistent with other studies that have demonstrated that women with higher levels of education have greater odds of experiencing SDM than women with less education because they are more self-assured and inquisitive [23–27, 31]According to a qualitative study in Tanzania, less educated women are also less likely to participate in SDM because healthcare providers believe they won’t comprehend things clearly and quickly [22].

Similar to what has been reported in other areas, cost-sharing group women had lower odds of participating in SDM compared to private/insured women, though the relationship was not statistically significant [23, 26, 32]. The study also discovered that employed women were more likely to participate in SDM than women in the unemployed group in line with other studies [31, 32]. Due to their perceived low social-economic status, the cost-sharing and unemployed groups were less likely to be involved in decision-making. The results, however, differ from studies that found that low-income and unemployed patients received more information during consultations [25, 33]

Women who started the conversation about their delivery method were more apt to participate in SDM. The results are consistent with observational research that found that clinicians provided patients with more information when they inquired about their treatment [25]. The results concur with other reports from various regions [34–37]. According to a qualitative study conducted in Tanzania, doctors were less engaged with patients who were not active in communication [22].

Patients are more likely to feel confident and engaged in discussing their treatment options when they have the support and encouragement of their family. The study found that companion participation in the conversations during antenatal visits had a favorable effect on SDM consistent with other studies [26, 38, 39]. Similarly, a greater degree of SDM was found in the married group compared to the divorced group, indicating the influence of family on the patients’ care. These findings further highlight the importance of companion participation in antenatal care.

According to studies in various regions, patients’ familiarity with their clinicians has been found to have a substantial association with SDM. [26, 32, 40]. Being comfortable with the clinicians helps with communication, confidence in the clinicians, and the impression of a more well-defined treatment plan. Majority of the pregnant women reported feeling familiar with clinicians after being treated by the same clinician more than twice. This result suggests that by enhancing personal continuity of care in clinics, SDM will be promoted.

### Strength of the study

The study used quantitative approach which provides numerical data as a baseline for future comparison as studies in this area are limited in Tanzania. The use of three different regional referral hospital provided diversity among study population which increases the ability to generalize the study results.

### Implication to practice

The less involvement of patients in SDM revealed in this study will encourage practitioners to incorporate SDM in daily practice to improve quality of care. The findings will also enlighten them on how to improve the practice of SDM.

### Study limitation and mitigations

The study assessed patient’s perception of SDM and not actual provider behavior, which may include their self-report and recall bias. This was mitigated by asking patients to recall information they were given for the past six months.

### Conclusion

The practice of SDM on mode of delivery among pregnant women with prior caesarean has been found to be low despite promotion of patient centered care in medical practice. Therefore efforts are needed to improve the implementation of SDM. Encouragement of companion involvement during antenatal care, promotion of personal continuity of care which improve familiarity of patients to clinicians and encouragement of clinicians to involve socially vulnerable population are recommended.

## References

[1] Betran AP, Ye J, Moller B, Souza JP. Trends and projections of caesarean section rates: global and regional estimates. 2021;1–8. 10.1136/bmjgh-2021-005671 PMC8208001 34130991

[2] Yaya S, Uthman OA, Amouzou A, Bishwajit G. Disparities in caesarean section prevalence and determinants across sub-Saharan Africa countries. 2018;1–9. 10.1186/s41256-018-0074-y PMC6027740 29988650

[3] Cavallaro FL, Pembe AB, Campbell O, Hanson C, Tripathi V, Wong KLM, et al. Caesarean section provision and readiness in Tanzania: analysis of cross-sectional surveys of women and health facilities over time. BMJ Open [Internet]. 2018 Sep 4;8(9):e024216. Available from: http://bmjopen.bmj.com/lookup/doi/10.1136/bmjopen-2018-024216 30287614 10.1136/bmjopen-2018-024216 PMC6173245

[4] Muganyizi P, Kidanto H, Kazaura M, Massawe S. Caesarean section: trend and associated factors in Tanzania. Afr J Midwifery Womens Health [Internet]. 2008 Apr;2(2):65–8. Available from: http://www.magonlinelibrary.com/doi/10.12968/ajmw.2008.2.2.65

[5] Litorp H, Kidanto HL, Nystrom L, Darj E, Essén B. Increasing caesarean section rates among low-risk groups: a panel study classifying deliveries according to Robson at a university hospital in Tanzania. BMC Pregnancy Childbirth [Internet]. 2013 Dec 8;13(1):107. Available from: https://bmcpregnancychildbirth.biomedcentral.com/articles/10.1186/1471-2393-13-107 23656693 10.1186/1471-2393-13-107 PMC3655870

[6] Cunningham FG, Bangdiwala SI, Brown SS, Dean TM, Frederiksen M, Rowland Hogue CJ, et al. NIH consensus development conference draft statement on vaginal birth after cesarean: new insights. NIH Consens; vbac new insight [Internet]. 2010 Mar 10;27(3):1–42. Available from: https://academic.oup.com/jnci/article/67/3/741/950230/National-Institutes-of-Health-Consensus 20228855

[7] Gupta J, Smith G, Chondankar R. Royal College of Obstetrician and Gynaecologists (RCOG) Green-top Guideline No. 45: Birth After Previous Caesarean Birth. Green-top Guidel [Internet]. 2015;45(45):31. Available from: https://www.rcog.org.uk/globalassets/documents/guidelines/gtg_45.pdf

[8] The American College of Obstetricians and Gynecologist. Vaginal Birth After Cesarean Delivery, Clinical Management Guidelines for Obstetricians-Gynecologists. ACOG Pract Bull [Internet]. 2019;205(184):1–25. Available from: https://journals.lww.com/greenjournal/fulltext/2019/02000/ACOG_Practice_Bulletin_No__205__Vaginal_Birth.40.aspx#pdf-link

[9] Pembe AB, Othman MK. Pregnancy outcome after one previous caesarean section at a tertiary university teaching hospital in Tanzania. Tanzan J Health Res [Internet]. 2010 Jun 30;12(3):188–94. Available from: http://www.ajol.info/index.php/thrb/article/view/53779

[10] Guise JM, Eden K, Emeis C, Denman MA, Marshall N, Fu RR, et al. Vaginal birth after cesarean: new insights. Evid Rep Technol Assess (Full Rep). 2010;(191):1–397. 20629481 PMC4781304

[11] Clark SL, Belfort MA, Byrum SL, Meyers JA, Perlin JB. Improved outcomes, fewer cesarean deliveries, and reduced litigation: results of a new paradigm in patient safety. Am J Obstet Gynecol. 2008;199(2):105.e1–105.e7. doi: 10.1016/j.ajog.2008.02.031 18468573

[12] Cox KJ. Providers’ perspectives on the vaginal birth after cesarean guidelines in Florida, United States: a qualitative study. BMC Pregnancy Childbirth [Internet]. 2011 Dec 12;11(1):72. Available from: https://bmcpregnancychildbirth.biomedcentral.com/articles/10.1186/1471-2393-11-72 21992871 10.1186/1471-2393-11-72 PMC3203084

[13] Lundgren I, Healy P, Carroll M, Begley C, Matterne A, Gross MM, et al. Clinicians’ views of factors of importance for improving the rate of VBAC (vaginal birth after caesarean section): A study from countries with low VBAC rates. BMC Pregnancy Childbirth [Internet]. 2016;16(1):1–10. Available from: 10.1186/s12884-016-1144-0 27832743 PMC5103375

[14] Leene HJJ. A declaration on the promotion of patient’ rights in Europe. Tijdschr voor Gezondheidsr [Internet]. 1994 May;18(5):100–6. Available from: http://link.springer.com/10.1007/BF03055676

[15] Schenker JG. Report of the FIGO Committee for the Study of Ethical Aspects of Human Reproduction. Int J Gynecol Obstet [Internet]. 1997 Jun;57(3):333–7. Available from: http://doi.wiley.com/10.1016/S0020-7292%2897%2902855-5 10.1016/s0020-7292(97)02855-5 9215501

[16] Scaffidi RM, Posmontier B, Bloch JR, Wittmann-Price R. The Relationship Between Personal Knowledge and Decision Self-Efficacy in Choosing Trial of Labor After Cesarean. J Midwifery Womens Health [Internet]. 2014 May;59(3):246–53. Available from: http://doi.wiley.com/10.1111/jmwh.12173 24850282 10.1111/jmwh.12173

[17] Gardner K, Henry A, Thou S, Davis G, Miller T. Improving VBAC rates: the combined impact of two management strategies. Aust New Zeal J Obstet Gynaecol [Internet]. 2014 Aug;54(4):327–32. Available from: http://doi.wiley.com/10.1111/ajo.12229 25117188 10.1111/ajo.12229

[18] Vankan E, Schoorel E, van Kuijk S, Nijhuis J, Hermens R, Scheepers H. The effect of the use of a decision aid with individual risk estimation on the mode of delivery after a caesarean section: A prospective cohort study. Solari A, editor. PLoS One [Internet]. 2019 Sep 26;14(9):e0222499. Available from: http://dx.plos.org/10.1371/journal.pone.0222499 31557177 10.1371/journal.pone.0222499 PMC6763212

[19] Biraboneye S P, Ogutu O, van Roosmalen J, Wanjala S, Lubano K, Kinuthia J. Trial of labour or elective repeat caesarean delivery:are women making an informed decision at Kenyatta national hospital? BMC Pregnancy Childbirth [Internet]. 2017 Dec 15;17(1):260. Available from: http://bmcpregnancychildbirth.biomedcentral.com/articles/10.1186/s12884-017-1440-3 28810843 10.1186/s12884-017-1440-3 PMC5558758

[20] Bernstein SN, Matalon-Grazi S, Rosenn BM. Trial of labor versus repeat cesarean: are patients making an informed decision? Am J Obstet Gynecol [Internet]. 2012 Sep;207(3):204.e1–204.e6. Available from: 10.1016/j.ajog.2012.06.057 22939727

[21] Chen SW, Hutchinson AM, Nagle C, Bucknall TK. Women’s decision-making processes and the influences on their mode of birth following a previous caesarean section in Taiwan: A qualitative study. BMC Pregnancy Childbirth. 2018;18(1):1–13. 29343215 10.1186/s12884-018-1661-0 PMC5773050

[22] Vedasto O, Morris B, Furia FF. Shared decision-making between health care providers and patients at a tertiary hospital diabetic Clinic in Tanzania. 2021;4:1–7. 10.1186/s12913-020-06041-4 PMC7780625 33397373

[23] Attanasio LB, Kozhimannil KB, Kjerulff KH. Factors influencing women’s perceptions of shared decision making during labor and delivery: Results from a large-scale cohort study of first childbirth. Patient Educ Couns [Internet]. 2018 Jun;101(6):1130–6. Available from: 10.1016/j.pec.2018.01.002 29339041 PMC5977392

[24] Leader C, Macphail S, Ayuk P, Robson S. VBAC or elective caesarean? a midwife-led shared decision making approach to choice following a caesarean section. Arch Dis Child—Fetal Neonatal Ed [Internet]. 2012 Apr 18;97(Suppl 1):A89.1–A89. Available from: http://fn.bmj.com/lookup/doi/10.1136/fetalneonatal-2012-301809.288

[25] Willems S, De Maesschalck S, Deveugele M, Derese A, De Maeseneer J. Socio-economic status of the patient and doctor–patient communication: does it make a difference? Patient Educ Couns [Internet]. 2005 Feb;56(2):139–46. Available from: https://linkinghub.elsevier.com/retrieve/pii/S0738399104000990 15653242 10.1016/j.pec.2004.02.011

[26] XU RH, Wong ELY. Involvement in shared decision-making for patients in public specialist outpatient clinics in Hong Kong. Patient Prefer Adherence [Internet]. 2017 Mar;Volume 11:505–12. Available from: https://www.dovepress.com/involvement-in-shared-decision-making-for-patients-in-public-specialis-peer-reviewed-article-PPA 28331297 10.2147/PPA.S126316 PMC5352249

[27] Chang H lan, Li F shan, Lin C fen. Factors Infl uencing Implementation Of Shared Medical Decision Making In Patients With Cancer. 2019;2019:13:1995–2005. 10.2147/PPA.S217561 PMC6885555 31819381

[28] Simon D, Loh A, Ha M, Kriston L, Scholl I, Ho L. Patient Education and Counseling The 9-item Shared Decision Making Questionnaire (SDM-Q-9). Development and psychometric properties in a primary care sample. Patient Educ Couns. 2010;80(1):94–9. 19879711 10.1016/j.pec.2009.09.034

[29] Renner RM, Eden KB, Osterweil P, Chan BK, Guise JM. Informational factors influencing patient’s childbirth preferences after prior cesarean. Am J Obstet Gynecol [Internet]. 2007 May;196(5):e14–6. Available from: https://linkinghub.elsevier.com/retrieve/pii/S0002937806021284 17466665 10.1016/j.ajog.2006.10.863

[30] Lazo-Porras M, Bayer AM, Acuña-Villaorduña A, Zeballos-Palacios C, Cardenas-Montero D, Reyes-Diaz M, et al. Perspectives, Decision Making, and Final Mode of Delivery in Pregnant Women With a Previous C-Section in a General Hospital in Peru: Prospective Analysis. MDM Policy Pract [Internet]. 2017 Jul 8;2(2):238146831772440. Available from: http://journals.sagepub.com/doi/10.1177/2381468317724409 10.1177/2381468317724409 PMC6125051 30288428

[31] Park SG, Derman M, Dixon LB, Brown CH, Klingaman EA, Fang LJ, et al. Factors Associated With Shared Decision–Making Preferences Among Veterans With Serious Mental Illness. Psychiatr Serv [Internet]. 2014 Dec;65(12):1409–13. Available from: http://psychiatryonline.org/doi/abs/10.1176/appi.ps.201400131 25178383 10.1176/appi.ps.201400131

[32] Menear M, Garvelink MM, Adekpedjou R, Perez MMB, Robitaille H, Turcotte S, et al. Factors associated with shared decision making among primary care physicians: Findings from a multicentre cross-sectional study. Heal Expect [Internet]. 2018 Feb;21(1):212–21. Available from: http://doi.wiley.com/10.1111/hex.12603 28768060 10.1111/hex.12603 PMC5750688

[33] NHS England. High quality care for all, now and for future generations: Transforming urgent and emergency care services in England—The Evidence Base from the Urgent and Emergency Care Review. 2013;1–79. http://www.england.nhs.uk/wp-content/uploads/2013/06/urg-emerg-care-ev-bse.pdf

[34] Edwards M, Davies M, Edwards A. What are the external influences on information exchange and shared decision-making in healthcare consultations: A meta-synthesis of the literature. Patient Educ Couns [Internet]. 2009 Apr;75(1):37–52. Available from: https://linkinghub.elsevier.com/retrieve/pii/S0738399108005260 19036550 10.1016/j.pec.2008.09.025

[35] Gordon HS, Street RL, Sharf BF, Souchek J. Racial differences in doctors’ information-giving and patients’ participation. Cancer. 2006;107(6):1313–20. doi: 10.1002/cncr.22122 16909424

[36] Street RL, Gordon H, Haidet P. Physicians’ communication and perceptions of patients: Is it how they look, how they talk, or is it just the doctor? Soc Sci Med. 2007;65(3):586–98. doi: 10.1016/j.socscimed.2007.03.036 17462801 PMC2811428

[37] Epstein RM, Feldman MD, Franz CE. Influence of Patients ‘Requests. JAMA psychiatry. 2014;293(16):1995–2003. 10.1001/jama.293.16.1995 PMC3155410 15855433

[38] Clayman ML, Roter D, Wissow LS, Bandeen-Roche K. Autonomy-related behaviors of patient companions and their effect on decision-making activity in geriatric primary care visits. Soc Sci Med [Internet]. 2005 Apr;60(7):1583–91. Available from: https://linkinghub.elsevier.com/retrieve/pii/S0277953604003879 15652689 10.1016/j.socscimed.2004.08.004

[39] Wolff JL, Roter DL. Family presence in routine medical visits: A meta-analytical review. Soc Sci Med [Internet]. 2011 Mar;72(6):823–31. Available from: 10.1016/j.socscimed.2011.01.015 21353358 PMC3070824

[40] Schers H. Familiarity with a GP and patients’ evaluations of care. A cross-sectional study. Fam Pract [Internet]. 2004 Nov 4;22(1):15–9. Available from: https://academic.oup.com/fampra/article-lookup/doi/10.1093/fampra/cmh721 10.1093/fampra/cmh721 15640289

